# DEFINING THE RELATIONSHIP BETWEEN ACTIVE CITIZENSHIP AND HEALTHY RURAL AGING: A CRITICAL APPROACH

**DOI:** 10.1093/geroni/igz038.063

**Published:** 2019-11-08

**Authors:** Rachel Winterton

**Affiliations:** La Trobe University, Bendigo, Victoria, Australia

## Abstract

Active citizenship is romanticized in policy for its role in keeping older adults healthy, and rural communities sustainable. However, as proportions of older adults resident in rural communities continue to increase, the gerontological literature has begun to highlight challenges associated with both the capacity and desire of rural older adults to be active citizens. Through an integrative review of the international literature, this paper interrogates how active citizenship trends among rural older adults support or hinder healthy aging in rural settings. Findings indicate that active citizenship among older adults can increase rural age-friendliness and facilitate individual wellbeing. However, practices associated with active citizenship among this cohort can disenfranchise certain groups of older adults, through reshaping societal norms relating to citizenship, age-friendliness and rurality. These findings indicate that programs designed to promote active citizenship must both consider, and account for, diverse capacities and desires for active citizenship among rural older adults.

